# Case report: Life threatening hyponatremia in infants with urinary tract infections: two cases of type III pseudohypoaldosteronism and review of the literature

**DOI:** 10.3389/fped.2023.1233205

**Published:** 2024-01-05

**Authors:** Cécile Carl, Lars Dinkelbach, Julia Mohr, Ruy Perez, Tobias Vera Lopez, Susanne Fricke-Otto, Tim Niehues

**Affiliations:** ^1^ Centre for Child and Adolescent Health, HELIOS Klinikum Krefeld, Krefeld, Germany; ^2^ Medical Faculty, RWTH University Aachen, Aachen, Germany; ^3^ Department of Pediatrics III, University Children's Hospital Essen, University of Duisburg-Essen, Essen, Germany

**Keywords:** pseudohypoaldosteronism, hyponatremia, hyperkalemia, urinary tract infections, hydronephrosis, congenital anomalies of the kidney and urinary tract

## Abstract

We describe two female infants at the age of five and six months with urinary tract infections presenting with vomiting and reduced drinking behavior. On laboratory analysis, severe hyponatremia (106 mmol/L and 109 mmol/L) was seen with hyperkalemia and compensated metabolic acidosis. Endocrinological analyses revealed massively increased levels of aldosterone and renin, leading to the diagnosis of type III pseudohypoaldosteronism (PHA). A review of the current literature 2013–2023 revealed 26 type III PHA cases aged up to ten months with reduced drinking behavior, weight loss and/or failure to thrive being the most common clinical presentations. Given the severe presentation of PHA electrolyte measurements in infants with urinary tract infections and/or in infants with congenital anomalies of the kidney and urinary tract (CAKUT) are strongly recommended.

## Background

Urinary tract infections (UTIs) in children are among the most common bacterial infections in childhood (1). Current guidelines suggest that oral treatment and thus outpatient care can be considered in infants older than 2 to 3 months of age (2). Severe complications are rare but include urosepsis and renal scarring (1). We present two cases of infants with UTI developing pseudohypoaldosteronism (PHA) and severe hyponatremia, a known but yet often unrecognized life threating complication of UTI in children less than 6 months of age (3). Further, a systematic review on the current literature is given.

## Case report

### Case 1

In September 2022, a five-month-old, previously healthy German female infant presented in our pediatric emergency room with a history of increased stool frequency and vomiting of four days. Physical examination revealed a pale child in reduced general condition, signs of moderate dehydration with a sunken fontanelle, mild tachycardia (129/min), a mildly prolonged peripheral capillary refill time of 2–3 s, dry lips, moist eyes without tears, dry nappies and cold extremities. The blood pressure on admission was 78/55 mmHg. Family history was unremarkable. The patient was admitted with the suspected diagnosis of gastroenteritis. Upon admission, blood gas analysis revealed severe hyponatremia (109 mmol/L), hypochloremia (81 mmol/L), hyperkalemia (8.5 mmol/L, venous serum 9.6 mmol/L) and compensated metabolic acidosis (pH 7.36; HCO_3_^−^ 15.5 mmol/L, pCO_2_ 22.2 mmHg, base excess −12.2 mmol/L). Repeated blood gas analyses as well as serum measurements confirmed these findings. Urine sodium was <20 mmol/L. Laboratory analyses revealed leukocyturia, erythrocyturia, nitrituria, an increased C-reactive protein (24.9 mg/L), leukocytosis (19.6 /nl) and thus the diagnosis of a urinary tract infection. Urine cultures were conducted which grew Enterobacter cloacae. Abdominal ultrasound showed a dilated right ureter, pyelectasis and a blurry differentiation between the renal medulla and cortex as an expression of inflammation.

### Case 2

The second case is a six-month-old, German, female infant who presented in January 2023 in the emergency room with a history of vomiting and reduced drinking behavior of three weeks. Previous history included a respiratory infection at the age of five months, a COVID-19 infection at the age of four months as well as failure to thrive (5,380 g corresponding to the 91th Percentile at the age of 7 weeks, 6,300 g corresponding to the 12th percentile at admission). Family history revealed a benign paroxysmal postural vertigo of the mother but was otherwise unremarkable. The clinical picture on admission is one of mild dehydration, characterized as sunken eyes, mildly prolonged capillary refill time (centrally 3 s, peripheral 2–3 s), mild tachycardia (151/min) and a blood pressure of 105/52 mmHg. Laboratory findings upon admission showed severe hyponatremia (106 mmol/L), hyperkalemia (7.0 mmol/L), hypochloremia (81 mmol/L) and a compensated metabolic acidosis (pH 7.41, HCO_3_^−^ 19.9 mmol/L, pCO_2_ 27.8 mmHg, base excess −5.2 mmol/L), an elevated C-reactive protein (59.1 mg/L) as well as leukocyturia, erythrocyturia, proteinuria and nitrituria. Urine cultures grew Klebsiella oxytoca. Urine sodium was 23 mmol/L. Abdominal ultrasound led to the finding of II°–III° hydronephrosis and enlargement of the right kidney.

### Treatment

In the light of life-threatening electrolyte deviations, both patients were admitted to the intensive care unit and initially treated according to the suspicion diagnosis adrenal insufficiency (4), receiving a hydrocortisone loading dose of 100 mg/m² followed by a parenteral hydrocortisone supplementation of 100 mg/m² over 24 h according to national guidelines (4). In Case 1, this was accompanied by fludrocortisone after the third day of treatment. Case 2 developed hypertension, most likely secondary to cortisol supplementation. Blood pressures spontaneously normalized (below the 95. Percentile for age) after day 8. Case 2 also had a short period of bradycardia (minimally 46 /min) on day 6, the electrocardiogram showed ventricular extrasystoles, echocardiography remained unremarkable.

Hydrocortisone and fludrocortisone were given for 6 days in Case 1. Hydrocortisone was given orally after day 5 and stopped after three additionally days in reduced dosage. Parenteral electrolyte supplementation to correct the decreased sodium levels were initiated directly after admission and slowly titrated under frequent controls (initially every two hours). Case 1 received 15.3 mmol/kg/d sodium parenterally while Case 2 required 8.6 mmol/kg/d sodium on day 1. In the following days, parenteral supplementation with 1–3 mmol/kg/d were required. In both cases, sodium levels normalized within three days after admission. A cerebral MRI in Case 1 on day 7 remained unremarkable, excluding central pontine myelinolysis (5). Potassium levels normalized within hours in both cases.

In both cases, an empiric parenteral antibiotic therapy with ampicillin and ceftazidim was initiated. Urine culture grew Enterobacter cloacae (Case 1) and Klebsiella oxytoca (Case 2) which led to the antibiogram-based therapy with piperacillin/tazobactam and ceftazidim. Antibiotics were consecutively given orally (sulfamethoxazole/trimethoprim and cefpodoxim) and stopped after 15 days (Case 1) and 9 days (Case 2) of treatment.

Hormone analyses, revealed massively increased aldosterone (>100 ng/dl) and renin (>500 µIU/ml) levels, indicating a reduced sensitivity against aldosterone and excluding adrenal insufficiency as the cause of the electrolyte deviations. ACTH values were within the normal range and cortisol levels were slightly increased in both cases (case 1: 21.2 µg/dl (measured at 3 a.m.) and case 2: 30.8 µg/dl (measured at 16 p.m.)), in line with a stress response to the patients’ severe infections. In combination with urine tract infections and structural abnormalities of the kidneys and ureters in both cases, the diagnosis of PHA type 3 was made. Hydrocortisone and fludrocortisone supplementation was reduced and stopped after 5 days in Case 1 and 8 days in Case 2, without recurrence of electrolyte deviations. Further hormone analyses (Division of Pediatric Endocrinology and Diabetes, Christian-Albrechts-University Kiel, Head: Prof. Dr. P.-M. Holterhus) showed increased levels of the steroid precursors 11-desoxycortisone and corticosterone, confirming the diagnosis.

**Figure 1:**
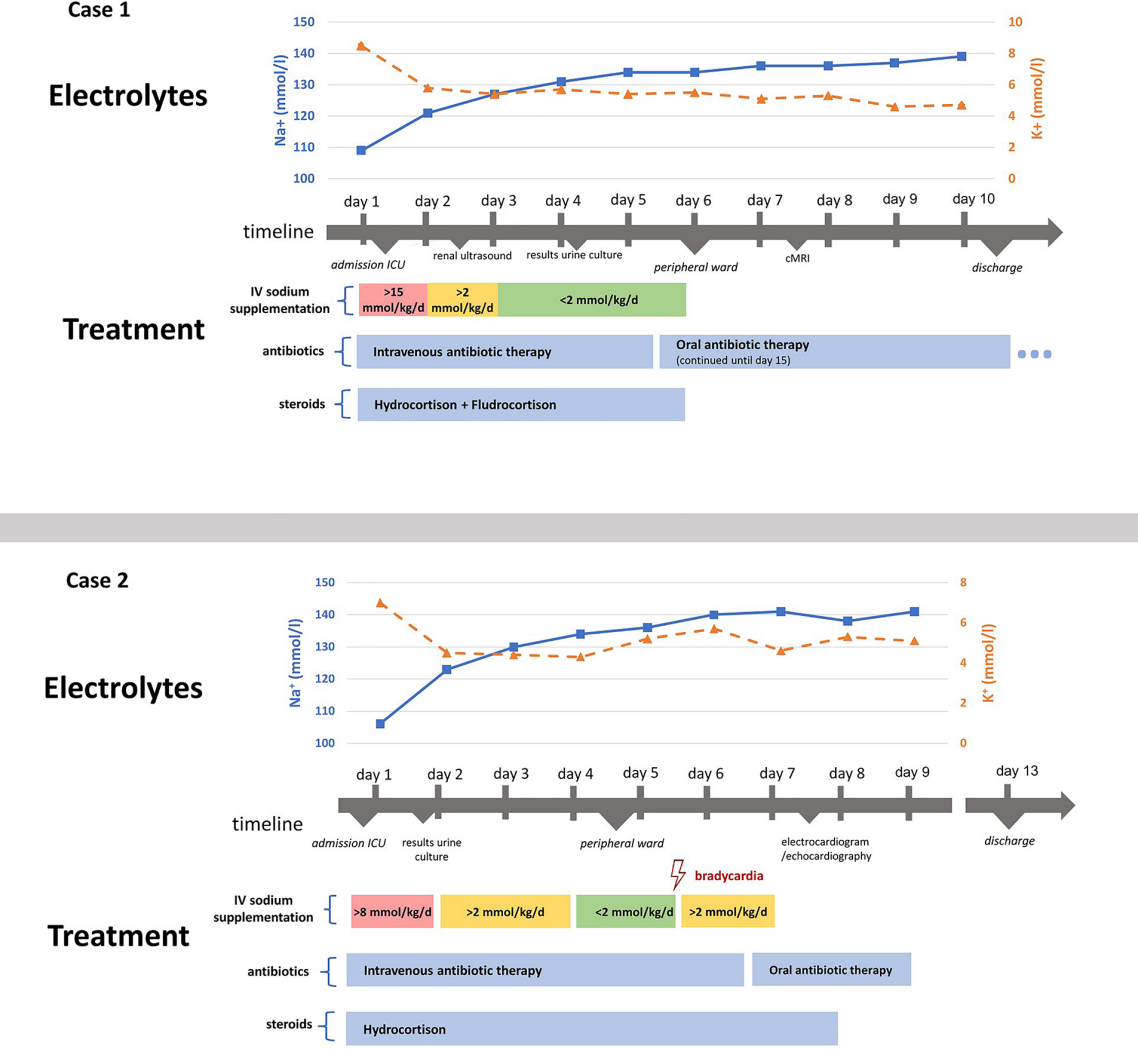
Illustration of both cases’ clinical course and treatment. Depicts the clinical course and treatment of both cases. Intravenous sodium supplementation is presented as mmol/kg/d, meaning the sodium amount in mmol given per kilogram body weight per day, summarizing different intravenous fluids given, including normal saline or balanced electrolyte solutions.

Both infants could be discharged 10 days (Case 1) respectively 13 days (Case 2) after admission. Figure 1 describes the clinical course during the hospitalisation with laboratory chemistry of the electrolytes and the initiated therapy of case 1 and 2.

Due to the sonographic findings, a voiding cystourethrography was conducted in Case 1 and revealed an ectopic megaureter and vesicoureteral reflux leading to III° hydronephrosis. The patient is currently receiving antibiotic prophylaxis with cefaclor and may undergo surgical correction in the future. Patient 2 is currently being followed as an outpatient in our pediatric nephrology department. In the age of 16 months, she was diagnosed with developmental delay and muscular hypotonia and is currently undergoing further neuropediatric and genetic diagnostics.

## Discussion

We present two cases of PHA type 3 secondary to urinary tract infections as complications of urinary tract malformations. These presented with very severe hyponatremia, hyperkalemia and massively increased aldosterone and renin levels. Correction of hyponatremia in both cases proved uncomplicated.

### Pathomechanism

Aldosterone causes the retention of sodium via epithelial sodium channels (ENaC) as well as the elimination of potassium via renal outer medullary K+ channels (ROMK), finally leading to hyponatremia and hyperkalemia. In PHA type 3, there is a resistance of aldosterone to renal mineralcorticoid receptors, leading to an electrolyte shift with hyponatremia, hyperkalemia and metabolic acidosis. It is closely associated with infections and/or congenital anomalies of the kidney and urinary tract (CAKUT) and mainly affects infants less than six months of age with a tendency of an increased risk in male infants (3). Recently, a case series described the occurrence of PHA type 3 in infants (mostly preterm) with high-output stomata, however the underlying mechanism and link to UTI or CAKUT-associated PAH type 3 remains unclear (6). Here, the combination of inflammation of the kidneys with its age-dependent immaturity appear to result in renal unresponsiveness for mineralocorticoids (7–10). The specific underlying mechanisms are not yet fully understood. One theory postulates that bacterial endotoxins damage the aldosterone receptor (10, 11). However, the presence of PHA type 3 with CAKUT without the presence of UTI, even representing a minority of cases, has repeatedly been reported (3, 10, 12). Another theory postulates that a rise in proinflammatory cytokines, either due to bacterial infection or increased intrarenal pressure, leads to an inhibition of renal mineralocorticoid receptors (10). However, neither of these mechanisms have been studied in detail. Additionally, it remains unclear why some infants suffer from those severe electrolyte alterations while most infants with urinary tract infections and/or CAKUT do not (7).

Additional yet unknown genetic alterations might contribute to the susceptibility of some infants. Tuoheti et al. conducted genetic testing in two cases with PHA and identified several heterozygote variants with unknown clinical significance (10). Future studies should systematically address potentially underlying genetic alterations which might help to identify infants at risk as well as to clarify potentially underlying pathomechanisms.

### Review of the literature

A systematic literature search of further case reports on transient pseudohypoaldosteronism was conducted in order to compare symptoms, diagnosis, therapy, and outcome of our two patients with previously published cases of pseudohypoaldosteronism type 3 and to update the comprehensive review by Delforge et al. (3). We searched PubMed for case reports that contained the term “pseudohypoaldosteronism” in their full text. To focus on the latest developments of the clinical picture, especially regarding therapy, the literature search was restricted to the period between 2013 and 2023. Only case reports published in English were considered. The last update of the literature search was 31.07.2023. In total, 100 papers were found and titles and abstracts were further screened for cases of type 3 pseudohypoaldosteronism, defined by transient pseudohypoaldosteronism secondary to UTI and/or hydronephrosis. In total, 17 case reports reporting on 26 cases of type 3 pseudohypoaldosteronism were found (8–10, 12–25). Table 1 depicts the clinical characteristics of the cases found in the literature search, together with our cases (*N* = 28).

**Table 1:** Review of the literature.

Author	*n*	Age	Symptoms	Laboratory findings (blood)	Urine	Comorbidities	Initial management	Outcome
Current report	2	5 months	vomiting, diarrhoea, poor feeding	Na^+^ ↓ (109 mmol/L), K^+^↑ (8.5 mmol/L), Cl^−^↓ (81 mmol/L), compensated metabolic acidosis, CRP↑, Leukocytosis, aldosterone↑ (>2,774 pmol/L), renin↑ (>300 ng/L)	leukocyturia, erythrocyturia and nitrituria, culture (Enterobacter cloacae)	right ectopic megaureter, VUR, HN III° (US/MCUG), UTI	fluids + electrolytes (F + E), antibiotics (AB), hydrocortisone (HC), fludrocortisone (FC)	
		6 months	vomiting, poor feeding, failure to thrive	Na^+^↓ (106 mmol/L), K^+^↑ (7.0 mmol/L), Cl^−^↓ (81 mmol/L), compensated metabolic acidosis, CRP↑, aldosterone↑ (>2,774 pmol/L), renin↑ (>300 ng/L)	leukocyturia, erythrocyturia, proteinuria and nitrituria, culture (Klebsiella oxytoca)	right HN II°-III° (US), UTI, ventricular extrasystoles (ECG)	F + E, HC, AB	
Abraham et al.	5	31 days	vomiting, failure to thrive, poor feeding, lethargy	Na^+^↓ (119 mmol/L), metabolic acidosis, aldosterone↑ (33,400 pmol/L), renin↑ (16,680 ng/L), urea↑, creatinine	Na^+^, culture (Enterobacter)	UTI, bilateral HN, VUR III° (right) and IV° (left) (US/MCUG)	F + E, AB	
		32 days	vomiting, failure to thrive, poor feeding, lethargy, fever	Na^+^↓ (111 mmol/L), K^+^↑ (8.0 mmol/L), metabolic acidosis, lactate↑, aldosterone↑ (7,540 pmol/L), renin↑ (75.6 ng/L), cortisol↑, urea↑, creatinine↑	Na↑, culture (E. coli)	UTI (US)	F + E, AB, HC, FC	left nephroureterectomy
		1 months	fever, irritability	Na^+^↓ (128 mmol/L), K^+^↑ (6.4 mmol/L), lactate↑, aldosterone↑ (22,100 pmol/L), renin↑ (155.82 ng/L), cortisol↑, urea↑, creatinine↑, blood culture (Staphylococcus aureus)	culture (Staphylococcus aureus)	UTI, scarred left kidney (MAG-3), left VUR IV°–V°, right HN and dilatated ureter (US/MCUG)	AB	outcome not specified, left nephroureterectomy
		6 months	poor feeding, irritability, vomiting, failure to thrive	Na^+^↓ (109 mmol/L), K^+^↑ (6.6 mmol/L), compensated metabolic acidosis, aldosterone↑ (1,06,000 pmol/L), renin↑ (93,600 ng/L), urea↑	Na↑, culture (E. coli)	UTI, bilateral VUR I°, right scarred kidney with severe pelvicalyceal and ureteric dilatation to the level of the VUJ (US/MCUG/MAG-3 scan)	F + E, AB, oral salt (over 5 days), HC	bilateral ureteric re-implantation
		3 months	poor feeding, vomiting, failure to thrive	Na^+^↓ (125 mmol/L), K^+^↑ (5.7 mmol/L), metabolic acidosis, aldosterone↑ (7,050 pmol/L), renin↑ (628.8 ng/L), urea↑, creatinine↑	culture (E. coli)	UTI, VUR I° (right) and II° (left), bilateral megaureter (US/MCUG)	F + E, AB	normalisation of aldosterone within 4 months
Atmis et al.	1	2½ months	fever, vomiting, dehydration	Na^+^↓ (111 mmol/L), K^+^↑ (7.04 mmol/L), metabolic acidosis, aldosterone↑ (>4,161 pmol/L), renin↑ (>500 ng/L), urea↑, leukocytosis, thrombocytosis	leukocyturia, nitrituria, and bacteriuria	UTI, antenatal hydronephrosis, right multicystic dysplastic kidney, left ureterovesical junction (UVJ) obstruction	F + E, AB	correction of the obstruction
Bakar et al.	3	2 months	fever, failure to thrive	Na^+^↓ (119 mmol/L), K^+^↑ (6,4 mmol/L), Cl^−^↓ (91 mmol/L), metabolic acidosis, aldosterone↑ (15,428 pmol/L)	leukocyturia, bacteria, positive leukocyte esterase, culture (ESBL Klebsiella pneumonia)	posterior urethral valve, partial scarred kidney with right to left differential function (DMSA), UTI	F + E, AB	
		2 months	fever, foul-smelling urine	Na^+^↓ (116 mmol/L), K^+^↑ (7.0 mmol/L), Cl^−^↓ (89 mmol/L), metabolic acidosis	urine analysis, culture (Pseudomonas aeruginosa)	vesico-vaginal fistula, UTI, right HN (MCUG)	F + E, AB	correction of the vesico-vaginal fistula
		8 months	fever	Na^+^↓ (112 mmol/L), K^+^↑ (6.4 mmol/L), Cl^−^↓ (85 mmol/L), metabolic acidosis	culture (ESBL Enterobacter cloacae)	Posterior urethral valve, bilateral HN, UTI	AB, oral F + E	Five episodes of UTI reported; In 3/5 episodes he had electrolyte shift; each time normalization with AB and oral sodium^a^
Ceylan et al.	3	43 days	failure to thrive	Na^+^↓ (118 mmol/L), K^+^↑ (7,05 mmol/L), aldosterone↑ (>4,438.4 pmol/L)	leukocyturia, culture (Klebsiella pneumoniae)	UTI, left HN und dilatated ureter (US)	F + E, AB, HC until hormonal studies	
		39 days	failure to thrive	Na^+^↓ (109 mmol/L), K^+^↑ (6.07 mmol/L), aldosterone↑ (>4,438.4 pmol/L), ACTH stimulation test	leukocyturia, erythrocyturia, culture (Klebsiella pneumoniae)	UTI, left HN und dilatated ureter (US)	F + E, AB, HC and FC until hormonal studies	oral hydrocortisone and fludrocortisone (duration not specified), pyeloplasty
		38 days	Failure to thrive, poor feeding	Na^+^↓ (118 mmol/L), K^+^↑ (5.2 mmol/L), aldosterone↑ (10,069.62 pmol/L)	leukocyturia, culture (E. coli)	UTI, bilateral HN und dilatated ureter (US)	F + E, AB, oral F + E	
Delhikumar et al.	1	15 days	vomiting, poor feeding, lethargy, moderate dehydration	Na^+^↓ (100 mmol/L), K^+^↑ (>6 mmol/L), urea↑, creatinine↑, metabolic acidosis, aldosterone↑ (3,758.77 pmol/L)	culture (E. coli), Na^+^↑, K^+^ to Na^+^ Ratio↑	UTI, right HN (US)	F + E, oral Kayxelate, AB, HC, FC	Disconnection of steroids after normalisation of hormone status; NaCl, Kayxelate until the first year of life
De Clerck et al.^b^	1	3 months	failure to thrive, poor feeding	Leukocytosis, Thrombocytosis, Na^+^↓ (126 mmol/L), K^+^↑ (6.8 mmol/L), Cl^−^↓ (88 mmol/L), metabolic acidosis, aldosterone↑ (1,00,085.92 pmol/L), renin↑ (1641.2 ng/L), cortisol↑	Urine analysis	UTI, unilateral HN and dilatated ureter	F + E, AB	
Huebner et al.	1	8 days	puffy hands and feet, dehydration, livedo reticularis, diarrhoe	Na^+^↓ (102 mmol/L), K^+^↑ (8.2 mmol/L), urea↑, creatinine↑, metabolic acidosis, blood culture (Enterococcus faecalis)	/	posterior urethral valve, bilateral HN (US, MCUG)	AB, F + E, HC	correction of the posterior urethral valve
Kibe et al.	1	4 months	poor feeding, acute pallor, lethargy, hypothermia	Na^+^↓ (114 mmol/L), K^+^↑ (11.5 mmol/L), metabolic acidosis, aldosterone↑ (62,137.6 pmol/L), urea↑, creatinine↑, CRP↑, Hb↓, Leukocytosis, Thrombocytosis	Urine analysis, culture (E. coli)	Cholelithiasis, bilateral HN (CT/US), UTI, acute kidney failure, cardiopulmonary arrest	F + E, AB, Insulin/Glucose, Ca^+2^-polystyrene-sulfonate, catecholamine	normalisation of hormones after discharge
Kodo et al.	1	12 days	poor feeding	Na^+^↓ (134 mmol/L), K^+^↑ (6.2 mmol/L), aldosterone↑ (4,655.327 pmol/L), renin↑ (36,700 ng/L/h), cortisol↑	Na^+^↓	left HN (US)	F + E, initial HC, oral F + E	oral therapy until steroid urine profile normalised at six months of age, normalisation of aldosterone and renin regressed on day 18
Krishnappa et al.	1	10 months	failure to thrive	Na^+^↓ (103 mmol/L), K^+^↑ (6.0 mmol/L), metabolic acidosis, aldosterone↑ (8,552.242 pmol/L), transtubular K^+^ gradient↑	Na^+^↓, leukocyturia, nitrites, culture (E. coli)	duplicated kidney with an upper pole ectopic ureter and VUR (US), UTI	F + E, AB, FC	Nephroureterostomy
Kumar et al.	1	1½ months^c^	vomiting, failure to thrive	Na^+^↓ (117 mmol/L), K^+^↑ (7.1 mmol/L), metabolic acidosis, lactate↑, aldosterone↑ (41,600 pmol/L), renin ↑ (>330 ng/L), thrombocytosis, Hb↓, urea↑, CRP↑, cortisol↑	haematuria	congenital hydrometrocolpos with active bleeding, bilateral HN (US), UTI	F + E, initial HC und FC, salbutamol, blood transfusion, AB, interventional treatment of hydrometrocolpos	
Latt et al.	1	3 months	diarrhea, dehydration, afebrile seizure, palpable ballooned right kidney	Na^+^↓ (117 mmol/L), K^+^↑ (9 mmol/L), Cl^−^↓ (90 mmol/L), metabolic acidosis, aldosterone↑ (3,700 pmol/L), ACTH-Stimulation-test (to exclude CAH)	culture (Enterobacter)	right Double kidney, right VUR V° (US/MCUG), UTI	F + E, Ca^+2^-resonium, AB	correction of the structural anomaly (urethral implantation)
Luketich et al.	1	5 months	Failure to thrive, global hypotonia, vomiting	Na^+^↓ (117 mmol/L), K^+^↑ (6.1 mmol/L), Cl^−^↓ (88 mmol/L), metabolic acidosis, Leukocytosis, Thrombocytosis, CRP↑, AST↑, ALT↑, aldosterone↑ (20,666.3 pmol/L), renin↑ (48,710 ng/L/h)	culture (E. coli)	bilateral HN, UTI, abnormal ECG	F + E, AB	
Morisaki et al.	1	4 months	poor feeding	Na^+^↓ (114 mmol/L), K^+^↑ (9.3 mmol/L), Cl^−^↓ (88 mmol/L), metabolic acidosis, creatinine↑, urea↑, aldosterone↑ (1,18,449.8 pmol/L), renin↑ (>20,000 ng/L/h), cortisol↑	hematuria, pyuria, proteinuria, culture (GBS)	left HN, bilateral VUR V° (US, MCUG), UTI	F + E, Glucose/Insulin, initial HC, AB	falling aldosterone und renin at day 28
Sethi et al.	1	21 days	vomiting, lethargy, inadequate body gain since birth	Na^+^↓ (103 mmol/L), K^+^↑ (8.2 mmol/L), Cl^−^↓, metabolic acidosis, Leukocytosis, CRP↑, aldosterone↑ (6,102.8 pmol/L), renin↑ (31,000 ng/L/h), Cortisol↑	pyuria, proteinuria, culture (Klebsiella pneumoniae)	UTI, Bilateral HN (US), VUR V°(MCUG), chronic kidney disease (DMSA)	F + E, AB, Ca^+2^-gluconate, salbutamol, HC, FC	kidney transplant
Tuoheti et al.	2	2 months	poor feeding, failure to thrive, irritability, palpable resistance in the left lower abdomen	Na^+^↓ (114,3 mmol/L), K^+^↑ (6.54 mmol/L), Cl^−^↓ (86.8 mmol/L), metabolic acidosis, aldosterone↑ (11,697.486 pmol/L)	culture	posterior renal cystic lesions associated with left calyces, left HN, left tortuous dilation of the ureter, distal ureteral obstruction (US, MRU), Cholecystolithiasis (CT, US)	F + E, initial HC, FC, furosemide	discontinuation of HC and FC at 6 months of age; Normalization of electrolytes and aldosterone at 13 months of life
		7 months	vomiting, fever	Na^+^↓ (114 mmol/L), K^+^↑ (8,34 mmol/L), Cl^−^↓ (79 mmol/L), metabolic acidosis, aldosterone↑ (2,875.251 pmol/L), renin↑ (3,350 ng/L/h), cortisol↑, CRP↑	leukocyturia, bacteria, proteins, culture (Morganella)	UTI, VUR, bilateral HN, megaureter, urethral valve, cholecystolithiasis	F + E, insulin, AB	
Xu et al.	1	1 mo nths	poor feeding, failure to thrive, lethargy, projectile vomiting	Na^+^↓ (116 mmol/L), K^+^↑ (10.2 mmol/L), Cl^−^ ↓ (91 mmol/L), metabolic acidosis, cortisol↑^d^	proteinuria, leukocyturia, haematuria, leukocyte esterase, culture (GBS)	bilateral HN und dilatation of ureters, VUR (MCUG/US), UTI	F + E, AB, HC (5 days), Ca^+2^-gluconate, polystyrensulfonat	normal electrolytes on follow up at 1 and 4 months

This table includes a systematic review containing published case reports on PubMed about pseudohypoaldosteronism type 3 during the period 2013–2023. The clinical and laboratory features, as well as therapy and outcome, were summarized. We used SI units for the values of electrolyte and hormones (aldosterone and renin). Normal range of sodium is 135–145 mmol/L. Normal range of potassium is 3.5–5.0 mmol/L and normal range of chlorid is 95–105 mmol/L. Normal ranges of aldosterone in infancy is 138.5–2493 pmol/L and renin 3–60 ng/L respectively 130–1740 ng/L/H. However, the values may vary depending on the source. AB, antibiotics; CAH, congenital adrenal hyperplasia; d, days; DMSA, dimercaptosuccinic acid scan; ESBL, extended-spectrum beta-lactamases; FC, Fludrocortisone; F + E, fluids + electrolytes (including NaCl, Ca + 2gluconate, Na2CO3 or HCO3-); GBS, group B Streptococcus; HC, Hydrocortisone; HN, Hydronephrosis; US, ultrasound; m, months; MRU, magnetic resonance urography; MAG-3, mercaptoacetyltriglycine diuretic renography; MCUG, micturating cystourethrogram; VUJ, vesicoureteric junction; VUR, Vesicourethral reflux.

^a^ Time of normalization not specified.

^b^ No access to full article.

^c^ Birth after 35 weeks of pregnancy (corrected 10 days old).

^d^ Aldosterone and renin were not tested due to lack of adequate sample volume.

In 20 of 28 cases electrolyte deviations rapidly resolved after sodium supplementation within hours up to six days, one case relied on sodium supplementation and polystyrene sulfonate up to the age of one year and in another case normalization of electrolytes and aldosterone was noted in follow up at 13 months. Both cases are questioning the diagnosis of transient PHA. In 6 cases, time of normalization of electrolytes was not specified.

Most patients were less than three months old (*n* = 16, 57% of cases), 7 cases (25%) were between 3 months and 6 months and 5 cases (18%) were older than 6 months up to 10 months. Most common presenting symptoms were general and unspecific in nature like reduced drinking behavior, weight loss and/or failure to thrive in 21 of cases (75%, multiple responses allowed) followed by vomiting (*n* = 13, 46%) or diarrhea in three cases (11%).

Our cases add specific facets to the spectrum of PHA type 3. First, our patients are relatively old as only five of the cases in the literature were older than four months. Second, we report two female cases, however, a strong tendency for PHA type 3 in male patients was previously reported (3). Third, our patients had severe hyponatremia below 110 mmol/L which highlight the potentially hazardous nature of PHA type 3.

### Differential diagnosis and diagnostics

Hyponatremia, hyperkalemia and metabolic acidosis in infants always should rise the suspicion of adrenal insufficiency, e.g., due to congenital adrenal hyperplasia (CAH) with salt wasting syndrome or a form of primary hypoaldosteronism (e.g., Aldosterone-Synthase-deficiency). Even if 17-hydroxyprogesterone is covered in newborn screenings in multiple countries worldwide including Germany, patients with residual activity of 21-hydroxylase or other forms of adrenal enzyme deficiencies leading to salt wasting syndrome might be missed and thus an unremarkable newborn screening does not rule out CAH. PHA can be distinguished from adrenal insufficiency by an excess of aldosterone and renin with high ACTH levels as a result of missing negative feedback mechanisms due to resistance in mineralcorticoid receptors. Cortisol levels can be normal or elevated because of the stress-inducing underlying infection. An excess of 17-hydroxyprogesterone, the hallmark of CAH due to 21-hydroxylase deficiency, cannot be seen in PHA.

PHA type 3 occurs secondary to infections and/or CAKUT and is transient while PHA type 1 and 2 are autosomal dominant and recessively inherited. In PHA type 1, a further distinction is made between the renal (autosomal dominant) and the systemic (autosomal recessive) form (26, 27). In the generalized form, salt loss occurs in several organs, including the lung, colon, kidney, salivary- and sweat glands. Known genetic alterations lay in the genes for the subunits of ENaCs. Symptoms such as poor feeding and vomiting usually occur in the first weeks of life (26). Therapeutically, salt and fluid replacement with high sodium, ion exchange resins and low potassium diet is crucial (26). The renal form of PHA type 1 is the result of mutations in genes encoding for the mineral corticoid receptor and autosomal-dominantly inherited (26, 28). The clinical manifestation varies greatly but are, in general, milder. Vomiting, weight loss and dehydration occur in young infants. In later childhood, the children have no symptoms and show normal development. The prognosis of the renal form is good and therapy with sodium supplementation can usually be terminated in early childhood (26, 28). PHA type 2 [also known as Gordon's syndrome (29) or familial hyperkalemia and hypertension] is an autosomal-dominant inherited disease with the clinical hallmarks of hypertension, hyperkalemia and mild metabolic acidosis, but, in contrast to PHA type 1 and 3, without marked hyponatremia (30). Laboratory abnormalities can be detected as early as infancy, while hypertension may be detected two to four decades later (31). It responds well to treatment with thiazide diuretics (30). Underlying mutations vary but usually isoforms of the so-called with-no-lysine kinases (WNK) genes are affected (27, 31) which lead to alterations of the renal electrolyte exchange via NaCl cotransporter (NCC) and/or ROMK channels (27, 31). PHA type 1 or 2 should be considered in cases with unusual presentation e.g., congenital onset, absent trigger factors (UTI or CAKUT) or unsuccessful withdrawal from sodium supplementation as well as a suspicious family history and should be further evaluated via genetic testing.

In infants with severe hyponatremia and/or the suspected diagnosis of PHA an abdominal ultrasound is mandatory to screen for urinary tract malformations and to exclude adrenal hyperplasia. Urine analyses should be conducted to screen for possible urinary tract infections. Additionally, hypernatriuria might be seen as a result to the absent of aldosterone mediated resorption of sodium via renal ENaCs (32). In our cases, urine sodium was <20 mmol/L (Case 1, measured on day 2) or only slightly elevated (23 mmol/L, Case 2), however this might be the result of the severe hyponatremia and thus a general shortage of sodium in both patients.

Another possible differential diagnosis in patients with hyperkalemia and metabolic acidosis is hyperkalemic renal tubular acidosis (RTA) type 4 which is characterized by reduced acid and potassium secretion (33). However, RTA type 4 is characterized by hyperchloremia, while our patients showed hypochloremia. RTA type 4 is usually secondary to kidney damage in patients with diabetes, interstitial nephritis (e.g., Lupus nephritis) or sickle cell nephropathy (33) and goes along with hypoaldosteronism or aldosterone resistance (33, 34). In the latter case, RTA type 4 can be seen as the clinical result of PHA. In children, patients with genetically proven PAH type 1 and type 2 and RTA type 4 have been described (34).

Other differential diagnoses of infants with severe hyponatremia include but are not limited to acute kidney or cardiac failure, gastroenteritis, especially in the light of concomitant vomiting and failure to thrive, as well as the syndrome of inappropriate antidiuretic hormone (SIADH) e.g., secondary to respiratory syncytial virus (RSV) bronchiolitis (35, 36).

### Therapy

In patients with severe hyponatremia, hyperkalemia and metabolic acidosis and signs of dehydration, normal saline bolus should be administered for electrolyte and volume supplementation. If a urinary tract infection is suspected, empiric antibiotic therapy should promptly be initiated. Hormone analyses which confirm the diagnosis of PHA usually take some time, therefore we and others suggest treating all cases as acute adrenal insufficiency until proven otherwise, even if PHA is suspected (13). Thus, hormone supplementation with hydrocortisone and/or fludrocortisone according to common guidelines (4) should be initiated. In our literature review, most cases received hydrocortisone and/or fludrocortisone for initial treatment (see Table 1). If elevated aldosterone and renin confirm PHA, hydrocortisone and fludrocortisone can be discontinued. During therapy with hydrocortisone, attention should be paid to various side effects, for example hypertension or bradycardia, as described in case 2.

### Complications

Hyponatremia can lead to cerebral edema and/or seizures and is associated with increased mortality and morbidity (35). Rapid correction of hyponatremia can lead central pontine myelinolysis associated with spastic quadriparesis (5). Luckily, despite the extreme hyponatremia in our patients, none of these complications were seen and cerebral MRI in Case 1 remained unremarkable.

Case 2 developed bradycardia (min. 46/min) and ventricular extrasystoles during a time when electrolytes already had been normalized. Bradycardia and hypertension can be seen with steroid replacement (37). In addition, elevated aldosterone levels, as excessively seen in our patient, might foster dysrhythmias (38). In addition, prior hyperkalemia might have promoted these dysrhythmias.

## Conclusions

We report two cases of infants with PHA type 3 secondary to urinary tract infections who developed life-threatening hyponatremia (minimally 106 mmol/L). Clinicians should be aware of the possibility of severe electrolyte deviations in infants with symptoms as vomiting or food refusal and urinary tract infections. Electrolyte measurements in infants with urinary tract infections should be conducted with low threshold considering these unspecific symptoms. Hyponatremia then calls for an extensive diagnostic work-up, including hormone analyses and renal ultrasound as well as prompt treatment, including sodium supplementation under frequent electrolyte controls, supplementation with hydrocortisone as well as antibiotic treatment, if not already started. Further research is needed to recognize type III PHA earlier and better and to enlighten the underlying pathomechanisms as well as dynamics of electrolyte deviations to identify infants-at-risk and to guide recommendations for electrolyte monitoring in infants with urinary tract infections.

## Data Availability

The original contributions presented in the study are included in the article/Supplementary Material, further inquiries can be directed to the corresponding author.
